# Robot body self-modeling algorithm: a collision-free motion planning approach for humanoids

**DOI:** 10.1186/s40064-016-2175-8

**Published:** 2016-04-27

**Authors:** Ali Leylavi Shoushtari

**Affiliations:** Department of Computer Engineering, Shoushtar Branch, Islamic Azad University, Shoushtar, Iran

## Abstract

Motion planning for humanoid robots is one of the critical issues due to the high redundancy and theoretical and technical considerations e.g. stability, motion feasibility and collision avoidance. The strategies which central nervous system employs to plan, signal and control the human movements are a source of inspiration to deal with the mentioned problems. Self-modeling is a concept inspired by body self-awareness in human. In this research it is integrated in an optimal motion planning framework in order to detect and avoid collision of the manipulated object with the humanoid body during performing a dynamic task. Twelve parametric functions are designed as self-models to determine the boundary of humanoid’s body. Later, the boundaries which mathematically defined by the self-models are employed to calculate the safe region for box to avoid the collision with the robot. Four different objective functions are employed in motion simulation to validate the robustness of algorithm under different dynamics. The results also confirm the collision avoidance, reality and stability of the predicted motion.

## Background

Central nervous system (CNS) manages the human posture and gesture by driving and control the musculoskeletal system in a way that not only resolves the kinematic and kinetic redundancies but also takes advantage of those to reach high maneuverability (Rashedi et al. 2010). From engineering point of view the redundancy both in kinematic and kinetic levels is considered as a problem in terms of system analysis while at the same time, it is a privilege in sense of design (to create a high maneuverable system). Evaluation and studying the human CNS’s strategy in performing dynamic tasks such as lifting (Ayoub 1998; Hsiang and Ayoub 1994; Xiang et al. 2010a; Sitoh et al. 1993; Leylavi Shoushtari 2013), walking (Anderson and Pandy 2001a, b; Xiang et al. 2009), jumping (Babič et al. 2006), somersault (Blajer et al. 2007) and standing up (Mistry et al. 2010; Lord et al. 2002; Janssen et al. 2002) is considered as a source of inspiration to control, motion planning (Abedi and Leylavi Shoushtari 2012) and motion learning (Oztop et al. 2008) of humanoids. The main idea is that human CNS considers several dynamic and static criteria to perform those tasks. Therefore, if we can define the dynamic/static constraints appropriately and sufficiently, the generated motion would be as same as human movement. Optimal motion planning framework is considered as an appropriate solution since numerous physiologically meaningful terms such as postural stability, physiological energy consumed and body physical capability can be included in as constraints and/or cost functions (Ivaldi et al. 2012). Accordingly, Ivaldi et al. (2010) proposed an online motion planning and control method for reaching movement for humanoid robots and Lord et al. (2002) and Janssend et al. (2002) evaluate sit-to stand movement and Mistry et al. (2010) proposed an optimization-based solution for human-like motion planning for this task.

Manual human lifting task is an important operation in many industrial processes which subjected to motion simulation in this research. It could be performed by different techniques (Anderson and Chaffin 1986) where “squat” and “back lift” are most common ones which can be characterized based on kinematical data of lifter (Zhang et al. 2000). Space time optimization (Chang et al. 2001) and predictive dynamics (Xiang et al. 2010b) are efficient optimization-based strategies and were used to human posture prediction of this task (Ayoub 1998; Hsiang and Ayoub 1994; Xiang et al. 2010a; Sitoh et al. 1993; Chang et al. 2001; Cheng and Lee 2005). Collision-Avoidance is one of the key features for realization of simulated motion. Numerous methods are presented in order to avoid the collision in simulation process. Wang and Hamam (1992) take advantage of an optimization-based solution to solve the collision-avoidance problem while Sezgin et al. (1997) implement the same strategy (optimal motion planning) to propose a collision resolution for set of redundant robots. The former approach was about object collision while later method was subjected to robot–robot collision. Later on, Yang and Meng took different approach and proposed an artificial neural network (ANN)-based solution to reduce the computation burden and made the resolution appropriate for implementing in real-time process (Yang and Meng 2000).

To deal with self-collision issue for hyper redundant robots such as humanoids, researchers are executed the artificial potential field (APF) based approaches which result smooth trajectories (Sahara et al. 2004; Sugiura et al. 2006; Khatib 1986). Ohashi et al. (2007) implemented this strategy to recognize dynamic/static obstacles by feeding back the distance from the robot’s hand and obstacle and Dietrich et al. (2012) proposed a reactive, torque-based self-collision free algorithm could be integrated into a task hierarchy for mobile two hands robot. Guan et al. (2006) evaluated the feasibility of stepping over the barriers by humanoids and represented an adaptive motion planning approach. The result of feasibility analysis is integrated in an algorithm for motion planning of feet and waist of humanoid. In the same research, the procedure of walking over the barriers was investigated in a case that the projection of the total center of mass (CoM) of the humanoid was kept within the base of the support (BOS) (Stasse et al. 2009). Yoshida et al. (2008) represent an iterative method for 3D collision-free motion planning where in each iteration, the kinematics of collision and dynamic feasibility of generated posture were checked by the algorithm. Dalibard et al. (2009) solved this problem using a randomized method and under stability and physical capability and task constraints. While Khansari-Zadeh and Billard (2012) proposed a unified framework based on Dynamical System to guarantee the collision-free motion planning of robotic manipulator with convex shaped obstacles.

In the abovementioned research topics (collision/obstacle avoidance during walking), robot has no significant interaction with surrounded environment. In fact, having physical interaction with environment in tasks such as cooperative manipulation, could criticize the collision avoidance achievements. Nowadays, the concepts of self-awareness and self-modeling are considered as key features in active learning and adaptability with environment (Gold and Scassellati 2007; Martinez-Cantin et al. 2010). Generally, the approaches based on this concept a set several kinematical/dynamical pre-defined models are implemented together with a higher level controller that would be responsible to select one of them (based on state of the system) as system’s model to achieve subjected task/tasks. The researchers have been designed a resilient robotic system which can adapt itself with changes in its hardware (for example due to the damage) by taking advantage of self-modeling ability (Bongard et al. 2006). A set of different dynamical self-models are considered for the robots then the robot is asked to do numbers of actions. The main idea is to recognize the best model (autonomously by robot) which can explain the relation between actuation and sensory data collected from mentioned trials. In summary the self-modeling approaches have been integrated in robotics for three purposes: (1) Robot self-recognition, (2) Self-estimation of the kinematical model of robot and (3) Damage recovery (last three rows in Table 1).

**Table 1:** A short summary of relevant research studies together with highlighted features in columns

Study	Motion generation approach	Collision avoidance algorithm	Self-modeling method	Target task	Application
Ayoub (1998)	Optimization	Checking the collision of box with knee	–	Human manual lifting	Occupational biomechanics
Xiang et al. (2010a, 2010b)	Multi-objective optimization approach	Virtual body spheres (located at joints)	–	Human manual lifting	Human biomechanics
Anderson and Pandy 2001a, b; Anderson and Pandy (2001a, b)	Dynamic/Static computational optimization	–	–	Human normal walking	Human kinesiology
Xiang et al. (2009)	Optimization (incorporation of recursive Lagrangian dynamics with analytical Gradients)	A sphere filling algorithm is applied to avoid the collision of wrist with hip	–	Human walking (under external loads i.e. backpack)	Human motion prediction
Blajer et al. (2007)	optimization	–	–	Jumping	Humanoid robot
Mistry et al. (2010)	Mimicking kinematics of human movement	–	–	Sit-to-stand	Humanoid robots
Wang and Hamam (1992)	optimization	A computational geometry algorithm to compute the distance between the robot segments and object	–	Robotic manipulation	Motion planning of robotic manipulator
Sugiura et al. (2006)	Null-space optimization criteria	Artificial potential field method	–	Walking	Humanoid robots
Ohashi et al. (2007)	Linear Inverted Pendulum Mode (LIPM)	Arm force feedback (which acts as a function of the distance from robot to obstacle)	–	Walking	Humanoid robots
Gold and Scassellati (2007)	Mapping from motor activity to motion	–	Dynamic Bayesian model	Self-recognition	Social robotics
Martinez-Cantin et al. (2010)	Active learning algorithm	–	Recursive Least Squares (RLS) estimation	Estimating the kinematic model of a serial robot	Social robotics
Bongard et al. (2006)	Forward locomotion generation through self-model algorithm	–	Continuous dynamics Self-Modeling	Damage recovery	Autonomous robots

Table 1 represents a summary of the relevant works addressed before and highlights the main features of each study in columns. The columns 2, 3 and 4 are allocated to novelty points of each work which are as follow: (1) Motion generation approach, (2) Collision avoidance algorithm and (3) Self-modeling method. The works presented in first four rows (Ayoub 1998; Xiang et al. 2009, 2010a; Anderson and Pandy 2001a, b) show that the optimal motion planning approaches are capable to predict human movements and so, these methods are appropriate for motion planning and control of humanoids too (fifth and sixth rows). Nevertheless, collision avoidance is another issue to be considered in motion planning of humanoid robots which is addressed in rows 7, 8 and 9. However, the applied collision avoidance algorithms have two major problems to be implemented in optimization-based motion planning approaches: (1) These algorithms are not consistent with optimization frameworks i.e. artificial potential field (APF) (Sugiura et al. 2006; Khatib 1986) and arm force feedback (Ohashi et al. 2007), (2) Even though there exist collision avoidance algorithms fitted into these frameworks i.e. virtual body sphere (Xiang et al. 2009, 2010a; Anderson and Pandy 2001a, b), these are not feasible (since don’t consider the geometry of whole body). In this research study we are taking advantage of the bio-inspired concept of “self-modeling” to present a feasible collision avoidance algorithm which is consistent with optimization based motion generation framework.

This paper presents a self-modeling-based approach to be implemented in a standard optimal motion planning algorithm. The proposed scenario works based on a set of 12 predefined kinematical self-models of human body that covers all of the feasible postures in manual lifting task. The models actually are parametric mathematical functions which represent a “*virtual boundary*” of human body. Then, the self-modeling algorithm is integrated in an optimal dynamic motion planning of manual lifting task to avoid object collision/self-collision (Xiang et al. 2010b). The algorithm uses the Cartesian position of the joints to calculate minimum horizontal distance required to move the hand back to avoid penetration of the box to the “virtual boundary”. In fact, the collision avoidance is defined as an inequality constraint and is implemented in optimization-framework together with the other constraints such as range of motion of joint, initial and final position of hand, lifting constraint to enhance the realization of the predicted motion. The lifting task is simulated for four different objective functions subjected to be minimized.

## Kinematics and dynamics of the system

**Fig. 1:**
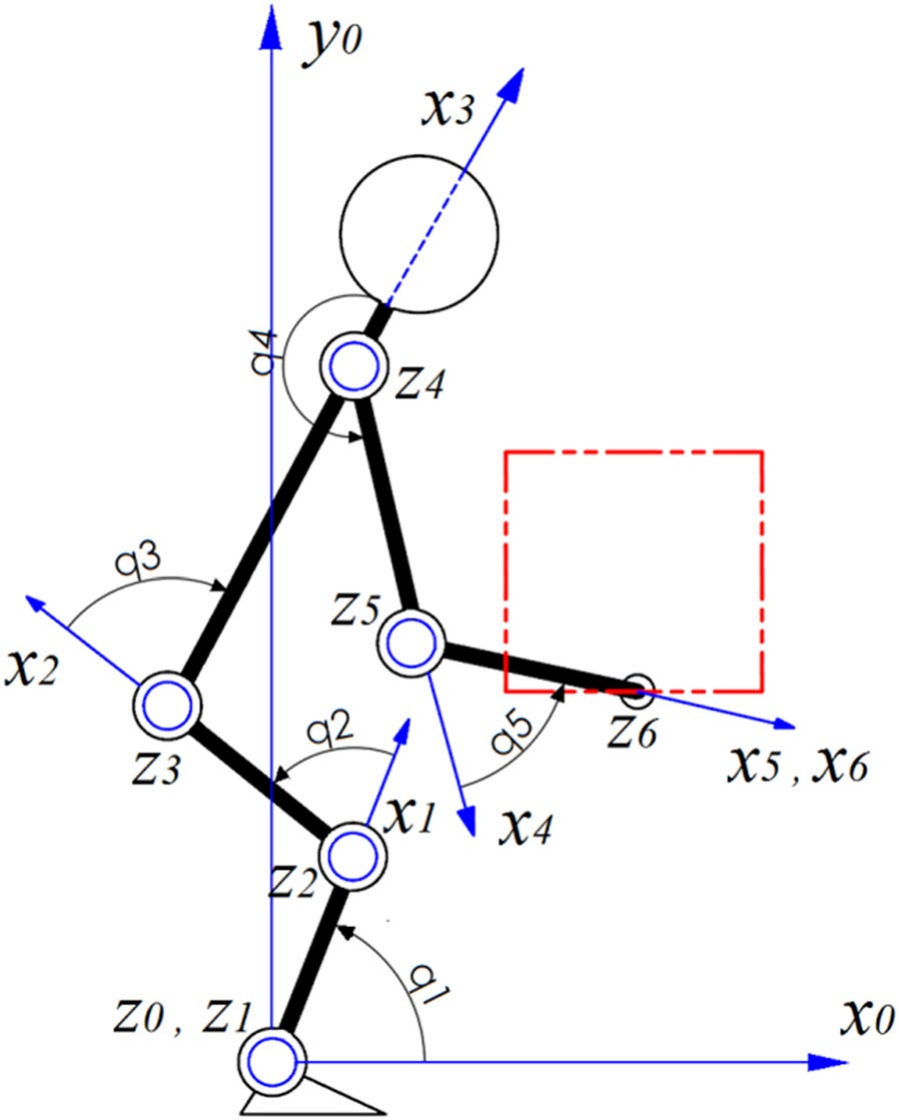
5DOF model of human body with coordination systems attached to each link

A biomechanical model with 5 DOF consists of 5 revolute joints in sagittal plane is used as a model of whole human body (Fig. 1). Human segments are considered as rigid bar with a mass point located in center of mass (COM) of each links. Forward kinematic and inverse dynamic equations of the system together with the kinematical and dynamical parameters of the model are addressed in Abedi and Leylavi Shoushtari (2012). The equations of motion which govern the dynamics of system is represented as (1). Where D(q) is 5 × 5 inertial and mass properties matrix, $C\left( {q,\dot{q}} \right)\dot{q}$ is Vector of coriolis and centrifugal forces, *V*(*q*) is gravitational force vector and *τ* is vector of external torques which consists of two parts: (1) joints produced torques by muscles *τ*_*muscle*_ and (2) torques exerted to joints due to box load *τ*_*box*_ obtained as (2). *J* is Jacobin transformation matrix of system and $m_{box} g^{T}$ is force vector due to the box weight.

1 $$D(q)\ddot{q}+C\left( {q,\dot{q}} \right)\dot{q}+V(q)=\tau$$

2 $$\tau = \tau_{muscle} + \tau_{box}\quad  ;\quad \tau_{box} = J^{T} \left( { m_{box} g^{T} } \right)$$

## Optimization-based motion simulation

**Fig. 2:**
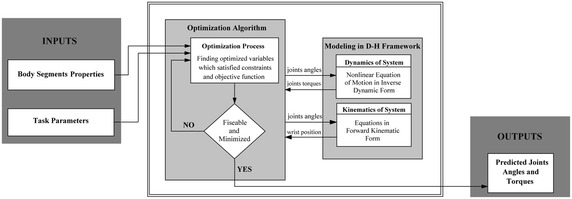
In unified simulation framework, the kinematical and dynamical equations are used by optimization algorithm as constraints. Body segments properties and parameters of task are used as inputs. The joint’s torques and angles which satisfied constraints and minimized the objective function are considered as output set

In this study an optimal motion planning approach (Leylavi Shoushtari 2013) is implemented to simulate manual lifting task. The approach uses inverse dynamics equation as an equality constraint to consider the dynamics of subjected motion in simulation process. The variables to be optimized are joint angles and torques. The kinematical and dynamical properties of human body and the parameters of motion task such as initial and final position, total lifting time are considered as inputs and the joint angles and torques make the output of the optimization-based algorithm. The algorithm has employed collision avoidance algorithm as an inequality constraint to check distance of the box with body. Figure 2 illustrate the schematic of this algorithm.

### Objective functions

Four different objective functions are defined in order to evaluate the performance of the collision avoidance algorithm and the reality of the predicted postures. The first function is designed based on the elastic property of the human joints which tend to maintain joints in an equilibrium point (midpoint respect to the lower boundary and upper boundary of joints range of motion). Considering $\theta_{{ub_{i} }}$ and $\theta_{{lb_{i} }}$ respectively as upper and lower boundary of range of motion and $\theta_{{ref_{i} }}$ as equilibrium point all for of *i*’*th* joint, the elastic energy will results as (3). For simplification the elastic coefficient *K* is considered as 1. So the elastic energy for whole human body (for 5 joints) during total lifting time *T* will obtain by (4). Due to the previous study ankle torque and total moment arm (TMA) of body segments (links of the model) could be consider as stability criteria during human movement (Abedi and Leylavi Shoushtari 2012). So to guarantee the stability of the movement two next objective functions are designed based on these parameters as (5) and (6). The last function is defined according to the fact that CNS tries to minimize metabolic energy consumed during movements (7). Where *τ*_*i*_ is the torque and *τ*_*i*_^2^ is metabolic energy of the *i*’*th* joint.

3 $$\theta_{{ref_{i} }} = \frac{{\theta_{{ub_{i} }} - \theta_{{lb_{i} }} }}{2}\quad ;\quad \varDelta \theta_{i} = \theta_{{ref_{i} }} - \theta_{i}\quad \to\quad E_{i} = \frac{1}{2}K\varDelta \theta_{i}^{2}$$

4 $$for\,\, K = 1\quad \to\quad F_{\varDelta \theta } = \frac{1}{2}\mathop \int \limits_{t = 0}^{T} \mathop \sum \limits_{i = 1}^{5} \varDelta \theta_{i}^{2} dt$$

5 $$F_{ank} = \mathop \int \limits_{t = 0}^{T} \tau_{ankle}^{2} dt$$

6 $$F_{TMA} = \mathop \int \limits_{t = 0}^{T} \left( {TMA} \right)^{2} dt$$

7 $$F_{\tau } = \mathop \int \limits_{t = 0}^{T} \mathop \sum \limits_{i = 1}^{5} \tau_{i}^{2} dt$$

## Collision checking through self-modeling scenario

The same constraints used in previous study (Abedi and Leylavi Shoushtari 2012) are integrated in this simulation. They briefly are as following: constraints used in this research are: joints torques and angles limitations, initial and final position of box, elevating constraint, inverse dynamics, and body collision avoidance constraint and inverse dynamic equation. The collision avoidance is considered as an inequality constraints in optimization process to check the penetration of box into the body. It’s inequality constraint and defined as a term of required horizontal distance *d*_*x*_ which wrist should move to prevent collision box with the body.

8 $$X_{bounndary} - X_{{wrist_{d} }} < 0$$

9 $$X_{bounndary} = X_{{wrist_{pr} }} + d_{x}$$

**Fig. 3:**
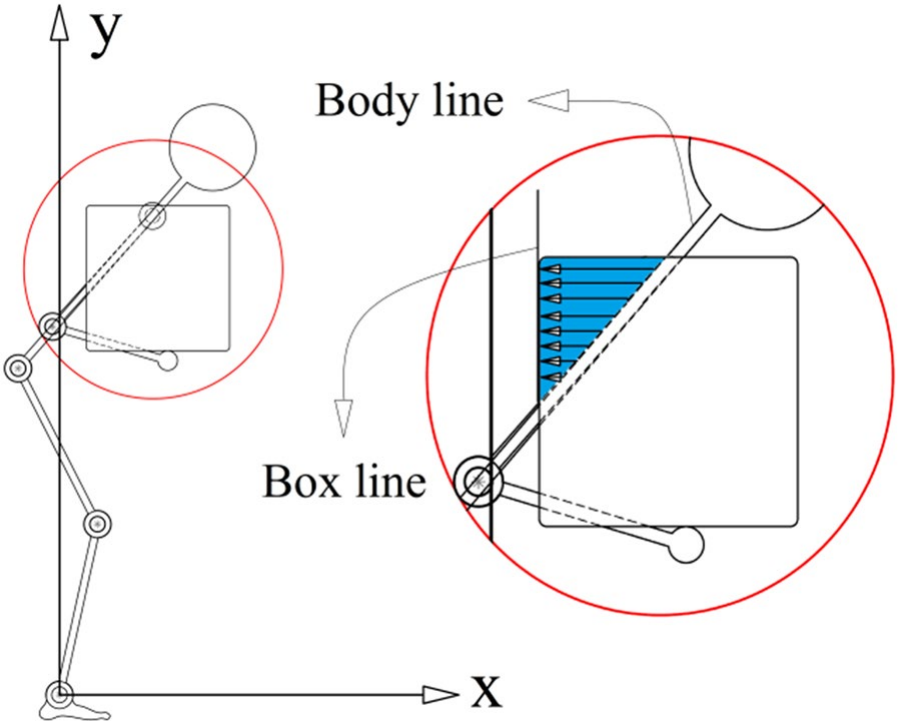
Penetration of the box into the body, *box line*, *body line* and penetration aria (with *blue colour*)

*d*_*x*_ is distance which wrist would move horizontally to arrive to the “boundary position” *X*_*boundary*_, and boundary position is a horizontal position of wrist where box edge would touch the body. $X_{{wrist_{d} }}$ is desire horizontal position of wrist which should be greater than *X*_*boundary*_ to avoid the collision. $X_{{wrist_{pr} }}$ is horizontal position of wrist obtained from optimization algorithm in each iteration. According to Fig. 3, penetration value of the box into the body *d*, *X*_*body*_ and *X*_*edge*_ would be obtained through Eq. (10), “body line” and “box line” respectively. *d* is penetration index so *d*_*x*_ is maximum value of *d* (11).

10 $$d = X_{body} - X_{edge}$$

11 $$d_{x} = MAX\left( d \right)$$

**Fig. 4:**
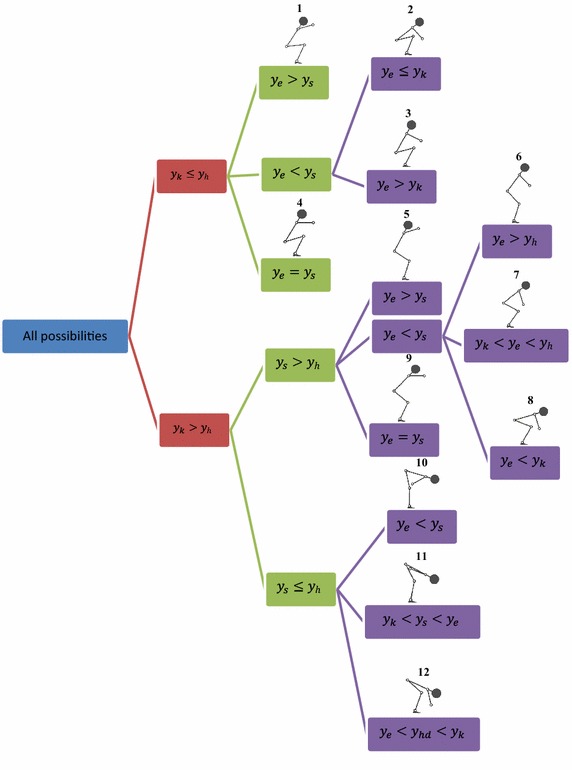
Possibility tree of body postures among manual lifting task. *Each branch* shows one feasible posture

**Fig. 5:**
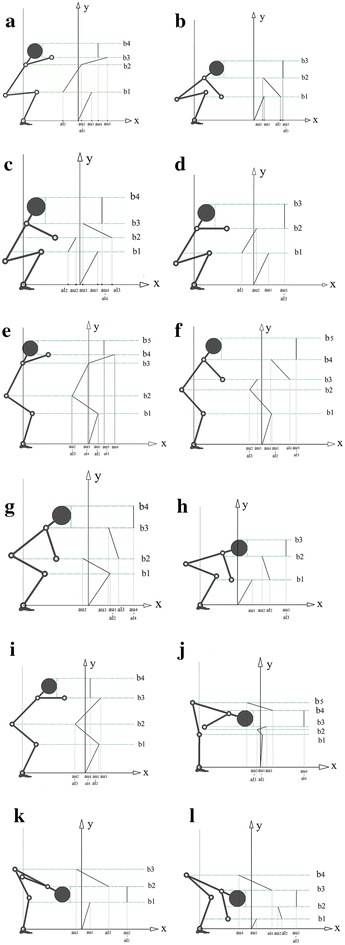
**a**–**f** The first 6 candidate self-models parametrically designed based on the vertical position of joints. **g**–**l** The second 6 candidate self-models parametrically designed based on the vertical position of joints

Now the problem is to find body position $X_{body} . \left[ {X_{body} , Y_{body} } \right]$ is a demonstration of position of boundary line calculated from configuration of the links. The position of “boundary line” is calculated from 12 candidate self-models; $j = 1, 2, 3, \ldots ,12$ related to 12 possible configuration specified by relative vertical position of joints (Table 1). The possibilities tree of human posture during manual lifting is illustrated in Fig. 4. This Figure shows all feasible postures out of possible joint arrangement in terms of relative vertical position of joints. Ankle is not considered in the possibility tree since it is assumed fixed to ground so ankle always is lower than all joints. Wrist is excluded because box never can collide with forearm consequently there’s no need to consider wrist position (Note that for constructing any link, we need to the position of two joints at the tips of the given segment i.e. for constructing the forearm we need to position of wrist and elbow). Accordingly, the variables ${\text{y}}_{\text{k}}$, ${\text{y}}_{\text{h}}$, ${\text{y}}_{\text{s}}$, ${\text{y}}_{\text{e}}$ and ${\text{y}}_{\text{hd}}$ presented in Fig. 4 are stand for vertical position of knee, hip, shoulder, elbow and COM of head respectively. The sign & is stand for “logical AND”. Figure 5 illustrates the 12 candidate functions together with their parameters. The function *F*_*j*_ specified as (13). *n* is number of the intervals defined in each configuration, *r*_*i*_ is the ramp of boundary line in each interval, *u*(*b*_*i*_) is an step function to determine intervals related to *Y*_*body*_. *al*_1_ and *b*_0_ has 0 values in Fig. 5.

12 $$X_{body} = F_{j} \left( {Y_{body} } \right)$$

13 $$F_{j} = \mathop \sum \limits_{i = 1}^{n} \left( {\frac{{Y_{body} - b_{i - 1} }}{{r_{i} }} + al_{i} } \right) \cdot u\left( {b_{i} } \right)$$

14 $$r_{i} = \frac{{b_{i} - b_{i - 1} }}{{au_{i} - al_{i} }}\quad;\quad u\left( {b_{i} } \right) = \left\{ {\begin{array}{*{20}l} 1 \hfill &\quad {b_{i - 1} \le Y_{body} < b_{i} } \hfill \\ 0 \hfill & \quad {otherwise} \hfill \\ \end{array} } \right.$$

## Results

A set of similar optimization problem solving process have been employed in previous studies (Xiang et al. 2009, 2010a, b; Leylavi Shoushtari 2013; Chang et al. 2001) for motion prediction and motion planning purposes. In this study, the optimization problem is designed for 10 evenly distributed time segments. By considering 10 torques and angle values for each joint, we have 100 (10 × 2 × 5) variables to be optimized. The optimization process is designed as a nonlinear quadratic programming in Matlab M-file environment. In which, the nonlinear programming solver named “fmincon” has employed to find the optimal variables which minimize the designed objective functions. The solver takes advantage of a recursive numerical algorithm to find minimum of constrained nonlinear multivariable function using sequential quadratic programming (SQP) method. In general it follows the following optimization framework (15) in which,$x$ and *f*(*x*) are vector of optimization variables and the objective function. Accordingly, and *C*_*eq*_(*x*) and *C*(*x*) are stand for the nonlinear function of equality and inequality constrains respectively. *lb* and *lb* also represents respectively the lower and upper limits of optimization variables (Table 2).

15 $$Find\, min\, f\left( x \right)\,such\, that\,\left\{ {\begin{array}{*{20}c} {C\left( x \right) \le 0} \\ {C_{eq} \left( x \right) = 0} \\ {lb \le x \le ub} \\ \end{array} } \right.$$

**Table 2:** It shows the 12 possible conditions due to the vertical position of the joints and relevant 12 candidates self-models

No. candidate self-model	Conditions according to relative positions of joints
1	*y*_*k*_ ≥ *y*_*h*_ & *y*_*e*_ ≥ *y*_*s*_
2	*y*_*k*_ ≥ *y*_*h*_ & *y*_*e*_ < *y*_*s*_ & *y*_*e*_ ≤ *y*_*k*_
3	*y*_*k*_ ≥ *y*_*h*_ & *y*_*e*_ < *y*_*s*_ & *y*_*e*_ > *y*_*k*_
4	*y*_*k*_ ≥ *y*_*h*_ & *y*_*e*_ = *y*_*s*_ & *y*_*e*_ > *y*_*h*_
5	*y*_*k*_ < *y*_*h*_ & *y*_*e*_ ≥ *y*_*s*_ & *y*_*e*_ ≥ *y*_*h*_
6	*y*_*k*_ < *y*_*h*_ & *y*_*e*_ < *y*_*s*_ & *y*_*e*_ ≥ *y*_*h*_
7	*y*_*k*_ < *y*_*h*_ & *y*_*e*_ < *y*_*s*_ & *y*_*e*_ < *y*_*h*_
8	*y*_*k*_ < *y*_*h*_ & *y*_*e*_ < *y*_*s*_ & *y*_*e*_ < *y*_*k*_
9	*y*_*k*_ < *y*_*h*_ & *y*_*e*_ = *y*_*s*_ & *y*_*e*_ > *y*_*h*_
10	*y*_*k*_ < *y*_*h*_ & *y*_*k*_ ≤ *y*_*s*_ < *y*_*h*_ & *y*_*k*_ < *y*_*e*_ ≤ *y*_*hd*_
11	*y*_*k*_ < *y*_*h*_ & *y*_*s*_ < *y*_*h*_ & *y*_*hd*_ < *y*_*e*_ ≤ *y*_*h*_
12	*y*_*k*_ < *y*_*h*_ & *y*_*s*_ < *y*_*h*_ & *y*_*e*_ < *y*_*hd*_ ≤ *y*_*k*_

Numbering is according to relative vertical position of joints. Where $\varvec{y}_{\varvec{k}}$, $\varvec{y}_{\varvec{h}}$, $\varvec{y}_{\varvec{s}}$, $\varvec{y}_{\varvec{e}}$ and $\varvec{y}_{{\varvec{hd}}}$ are vertical position of knee, hip, shoulder, elbow and COM of head respectively. The sign & is stand for “logical AND”

The “task parameters” i.e. lifting time, weight and dimension of the manipulated object, initial and final position of the object and “body segments properties” i.e. length, mass, inertial properties and position of the center of mass of each body segment are used as inputs for this optimization algorithm. Then the kinematical and dynamical model of human body is employed as two main set of constraints using the mentioned input parameters. These two sets of constraints are responsible for checking the kinematic and dynamic consistency of the predicted movement. The designed objective functions are aimed to impose different dynamic on the predicted movement to check the efficiency of the collision avoidance algorithm under different dynamics.

**Fig. 6:**
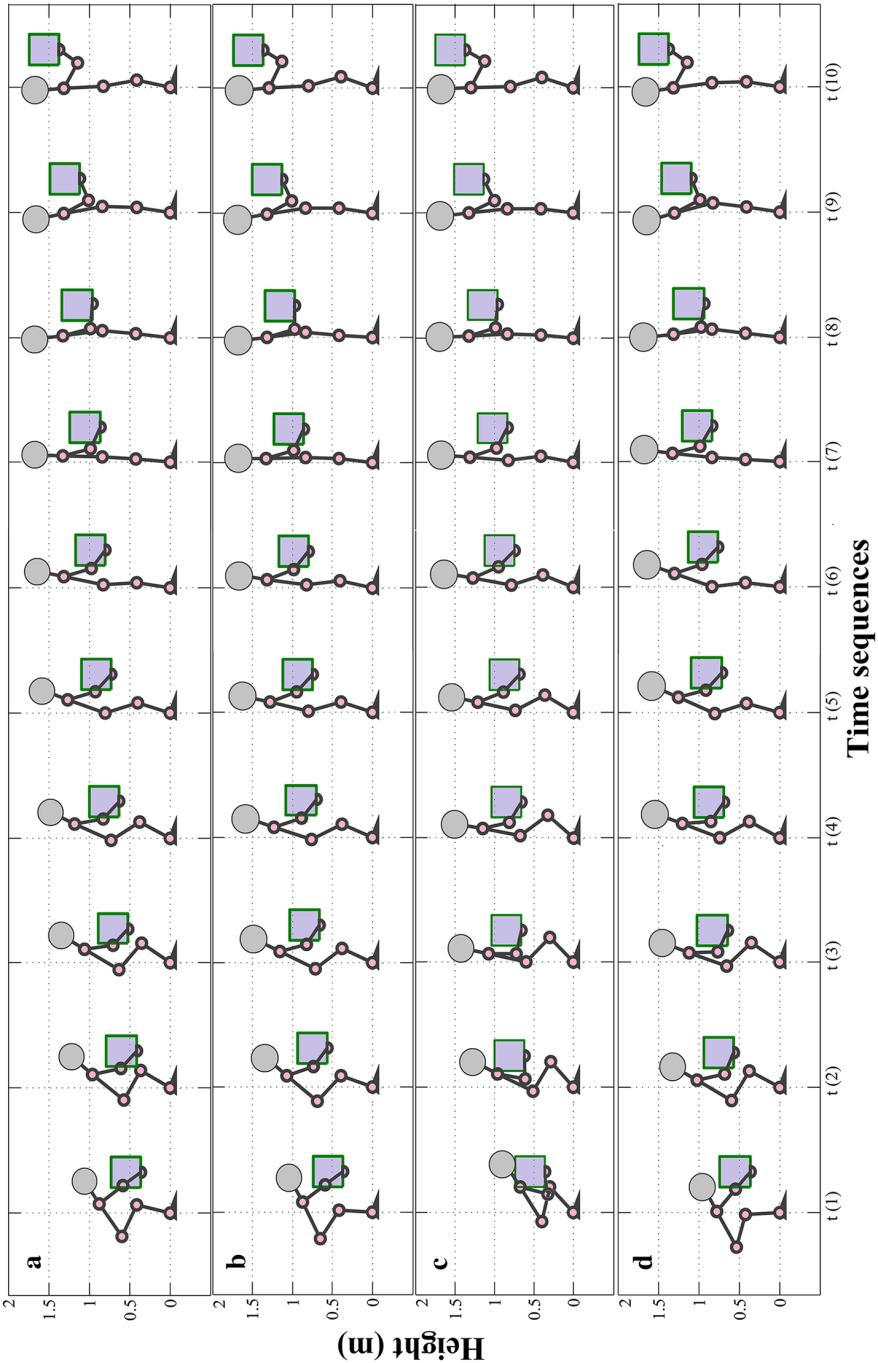
Snapshots of body postures during lifting task. The four different postures set are resulted in minimizing of following objective functions: **a** $F_{TMA}$, **b**. $F_{ank}$, **c**.$F_{\varDelta \theta }$ and **d**. $F_{\tau }$. The vertical axis is height in meter and the horizontal axis shows the time vector

**Fig. 7:**
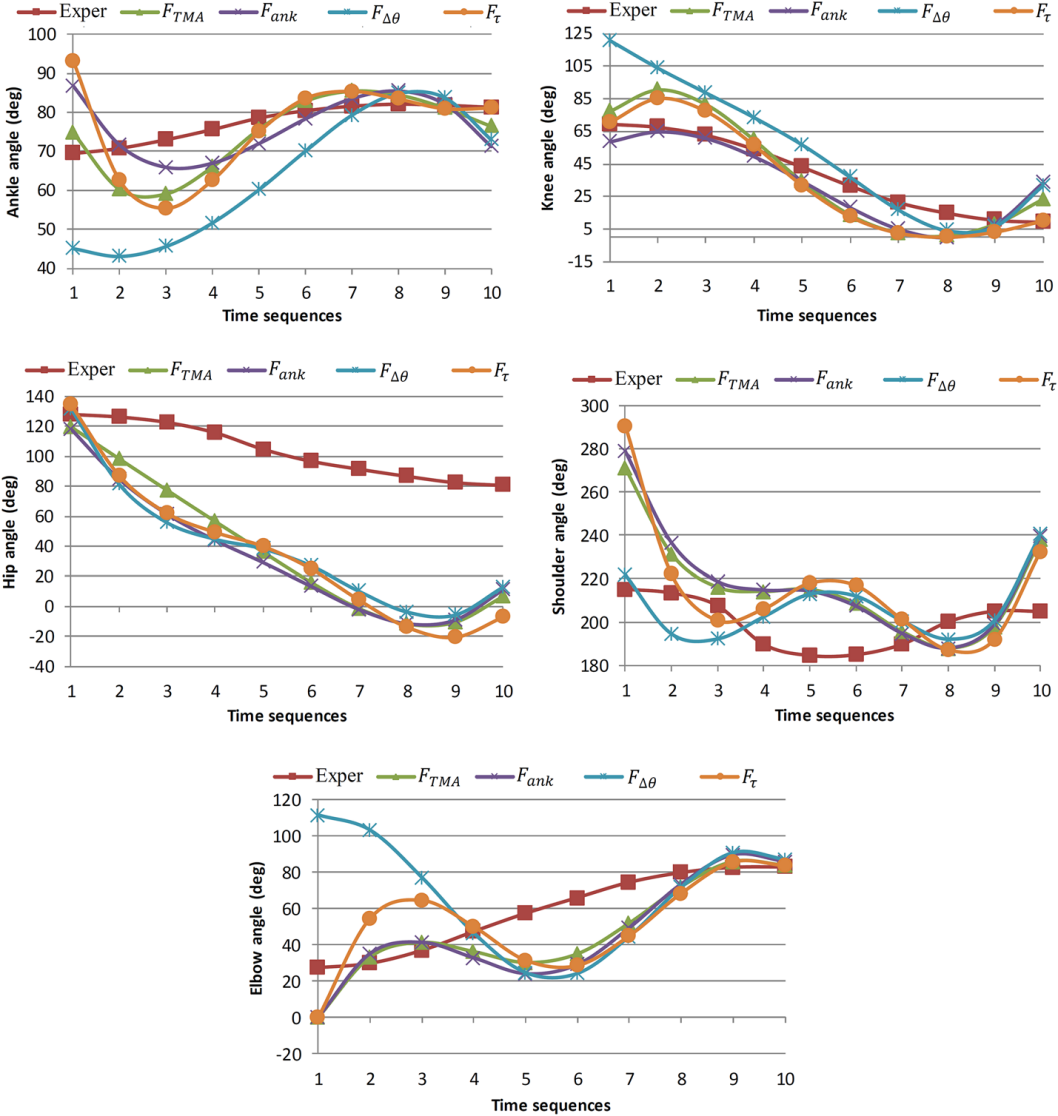
Predicted joints angles profiles in comparison with experimental results. Exper is stand for experimental data. TMA, ATsum, Dqssum and TrqSum are predicted joint profile resulted in minimization of following objective functions respectively: *F*_*TMA*_, *F*_*ank*_, $F_{\varDelta \theta }$ and *F*_*τ*_. The vertical axis is joints angles which are in degree and the horizontal axis shows the time sequences

**Fig. 8:**
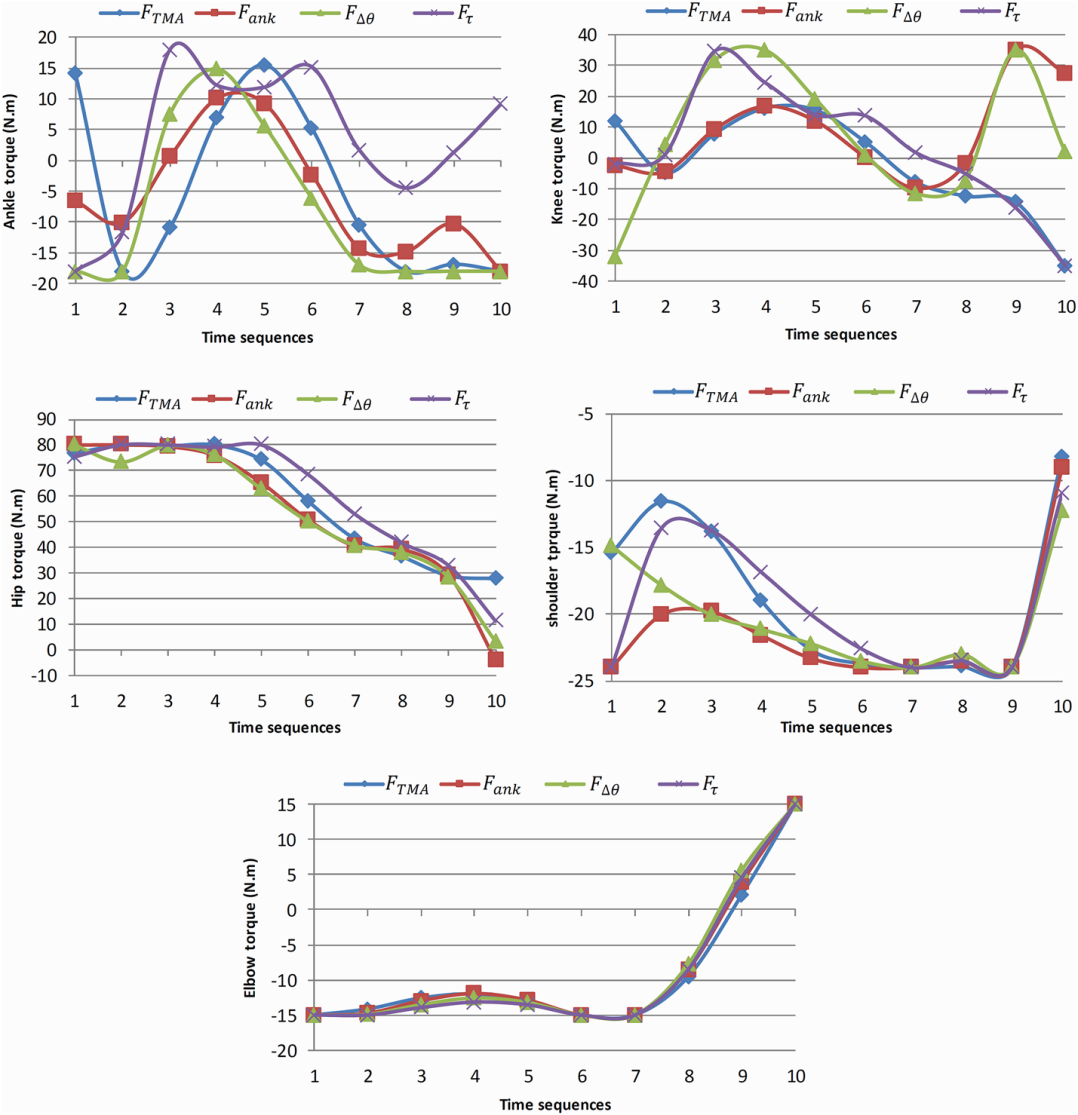
Torque profiles result in optimization process TMA, ATsum, Dqssum and TrqSum are predicted joint torques resulted in minimization of following objective functions respectively: $F_{TMA}$, $F_{ank}$, $F_{\varDelta \theta }$ and $F_{\tau }$. The vertical axis is joints toques which are in Newton. Meter and the horizontal axis shows the time sequences

Figure 6 shows the snapshots of the body postures during lifting time resulted in minimization of four mentioned objective functions. The experimental data of joint profiles used in (Xiang et al. 2010a) are implemented to validate this simulation result. In this research (Xiang et al. 2010a), an experiment on manual lifting task is carried out in which, the body motion of five healthy male subjects are captured and accordingly, angle profile of main human joints (ankle, knee, hip, shoulder and elbow) are extracted. The subjected population of experiment has characterized as follow: the average height and weight respectively are 1.7058 m and 64.8637 kg and the mean age of the participants is 34 years. The Lifting task parameters are presented in Table 3 and the predicted joint angles are plotted in Fig. 7. In this Figure optimal joint angle profiles are illustrated together with experimental results. The predicted torque profiles for all of the five joints are represented in Fig. 8. TMA which stands for stability criterion is plotted in Fig. 8 for the mentioned objective functions. The stability margins for TMA parameter are characterized by horizontal position of toe and heel placed at 0.183 and −0.078 m respectively.

**Table 3:** Lifting task parameters and values

Lifting parameters	Values
Box depth	0.370 m
Box height	0.365 m
Box weight	9 kg
Initial height	0.365 m
Final height	1.37 m
Initial horizontal position	0.490 m
Final horizontal position	0.460 m
Lifting time duration	1.2 s

## Discussion

In this research, the dynamic motion simulation of human lifting was planned as an optimization-based problem with 100 variables. Total lifting time was divided in 10 evenly distributed sequences. The first and last sequences are designed to be coincided with starting and finishing lifting time, so the initial and final postures are optimized by the algorithm and there’s no need to pre define these postures anymore. The novel body collision avoidance algorithm inspired from body self-awareness ability of human was implemented successfully in optimal motion planning framework. The basic idea is based on the automatic selection of body models with the respect to the posture predicted by the algorithm. Later, the model will be implemented in calculation of the desired position of robot’s wrist where box would not collide with the body. The lifting motion was generated using four biologically meaningful objective functions to evaluate the performance of the collision avoidance algorithm for motions with different dynamics. Finally the stability index TMA was measured for all of the four simulation result to demonstrate the stability of predicted motions.

Comparison between predicted and experimental results shows good compatibility in joint angle profile in Fig. 7 in terms of joint profile’s trends. In particular, the predicted profiles for joint angles have the same trend as the experimental profiles while their amplitudes are higher. Based on Fig. 7, the predicted profiles for the shoulder is not even have same curvature as experimental results which the un-modeled DOFs of the shoulder (this joint has 3DOFs) would be a reasonable explain for that. The predicted angles for three of objective functions ${\text{F}}_{\text{TMA}}$, ${\text{F}}_{\uptau}$, ${\text{F}}_{\text{ank}}$ almost are the same while for ${\text{F}}_{\Updelta \uptheta}$ it differs. Since the $F_{\Updelta \uptheta}$ is a kinematical-based defined function so it directly effects on the kinematics of the system (joint angles). So the kinematical nature of this objective function would be the reason of this difference. It is also recognizable in Fig. 6 by comparing posters set C with the other three sets. In particular, in posture set C the body starts motion with a completely squatted posture due to the foot dorsiflexion ($\theta_{ankle} = 45{^\circ }$) and knee flexion ($\theta_{knee} = 120{^\circ }$). While, the predicted motion for the other three objective functions initiate while the shank is almost vertical ($\theta_{ankle} \approx 45{^\circ }$) and knee is in extension mode ($\theta_{knee} \approx 70{^\circ }$). Consequently, the body requires to a forward bending in order to reach to the box. These two predicted modes of the lifting motion resemble two well-known lifting techniques i.e. leg lift or squat and back lift. In particular, the posture set C in Fig. 7 is similar to leg lift method since the body starts with the squatted posture and the rest of the posture sets (especially B and D since their initial postures of shank are quite vertical) looks like back lift technique due to the forward bending of the initial body postures.

According to the predicted angle profiles of joint, the lifting motion can be divided in two main portions: the primer starts from first to fifth time sequences and secondary is from the sixth to last one. The joints of lower body activate more (rather than lower one) within the former section while in second section the joints contributed to the upper body (specially elbow) activate more than the lower joints. This fact is clearly demonstrate by torque profiles of the ankle for three objective functions i.e. $F_{\Updelta \uptheta}$, *F*_*τ*_ and *F*_*ankle*_ in Fig. 8 where the torque deviation in first part of the motion (−20 to 15 N.m) is quite greater than the second part (−20 to −10 N m). Likewise, the forth torque profile in the first portion has a deviation of −20 to −20 N m while in the second part it is reduced to [5 N m 15 N m]. In the first section of motion of Fig. 7, the angle profiles of knee predicted for three objective functions i.e. *F*_*TMA*_, *F*_*τ*_ and *F*_*ankle*_ varies from 25° to 85° while in the second section it deviates from 0° to 25° Similarly, the forth angle profile in the first portion has a deviation from 25° to 120° while this amplitude reduces to 0° to 30° in the last part. In contract to the lower joints, the torque profiles of elbow presented in Fig. 8 have inverse trend where the predicted torque of the elbow in the first section is almost constant (−15 N m) while in the last part, it varies from −15 to 15 N m. In summary, the lower joints (ankle and knee) are active more in the first section of the motion rather than second portion. While, elbow has an inverse trend and it activates more in the second section of lifting time.

**Fig. 9:**
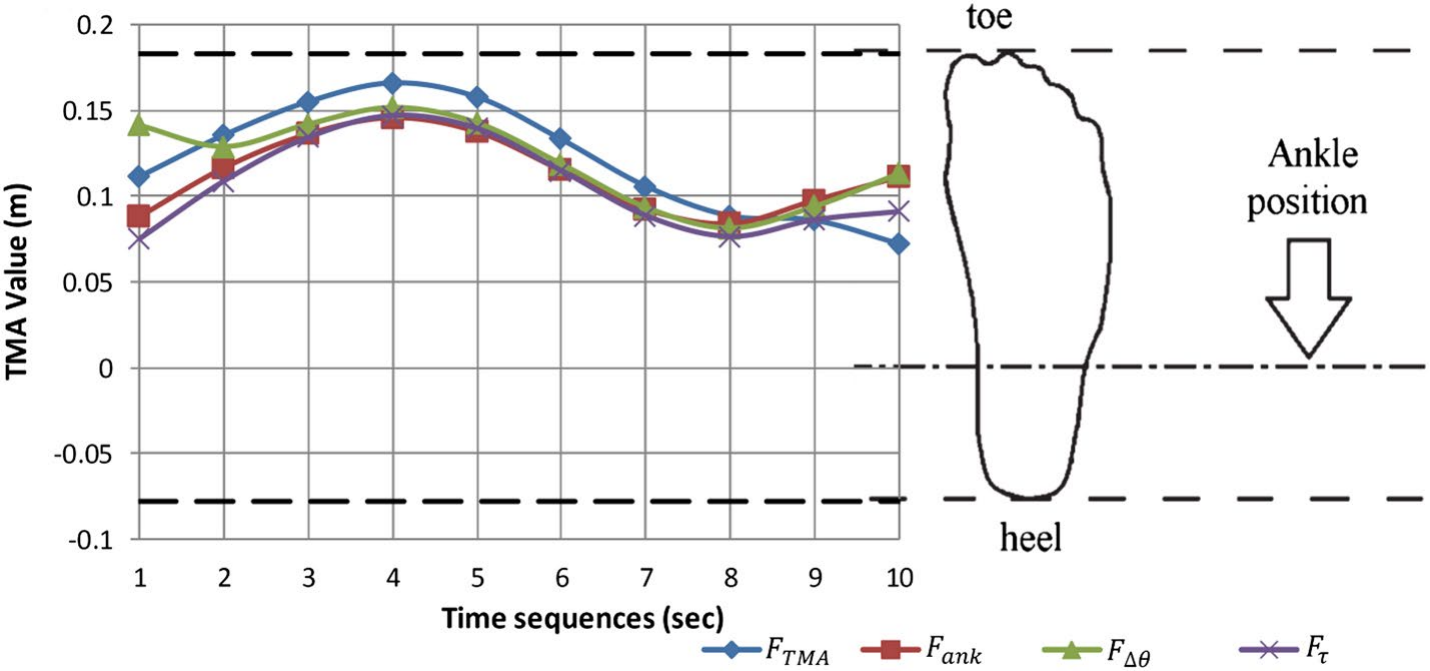
TMA values during lifting time resulted in minimization of four objective functions are illustrated together with toe and heel lines as boundaries of base of support (BOS). TMA, ATsum, Dqssum and TrqSum are predicted joint profile resulted in minimization of following objective functions respectively: $F_{TMA}$, $F_{ank}$, $F_{\varDelta \theta }$ and $F_{\tau }$. The bounded values of TMA prove the stability of the motion. The vertical axis is total moment arms of links which is in Meter and the horizontal axis shows the time sequences which is in 0.12 s scale

Since the initial position of the box is ahead and also lower than the position of the shoulder so, to get to the box, shoulder needs to be flexed i.e. the initial shoulder angles in Fig. 7 and elbow is required to be extended i.e. the initial elbow angles (Fig. 7). In the next time sequences, the shoulder gradually extends due to the upward motion of body and motion of the box toward the body. In the posture sets A, B and D in Fig. 6, we are witnessing that the box is pulled toward body within 2nd to 5th time sequences which can be explained by elbow flexion illustrated in elbow profile in Fig. 7. However, the predicted motions for third objective function ($F_{\varDelta \theta}$) has different story. Since the objective function tends to keep joints in the middle of the range of motion, we are witnessing that the motion starts with the flexion of the elbow while shoulder initiates the motion with an extended posture. In the next time sequences, the elbow gradually extends and the shoulder follows the same trend as other profiles. In fact, in the initial body posture predicted by this objective function $F_{\varDelta \theta}$, the body is close to the box while the TMA value (0.14 m) is near to margin of the stability (0.20 m) (Fig. 9). In other words, the body has started with a potentially unstable posture. Consequently, in next time sequences the body (or COM of body) moves backward in order to avoid this potential instability i.e. the second time sequence in Fig. 9 in which the TMA value for $F_{\Updelta \uptheta}$ reduces to 0.12 m. Hence, from the beginning to the end of the first portion of the lifting (from 1st sequence to 5th), the body moves away from the box which consequences the elbow extension.

At the beginning of the second portion of movement (sequence 5), the body is in upright posture and the box has a distance from body which will move the system to an unstable region. This fact is demonstrated in Fig. 9 where the TMA value (≈0.15 m) is close to the margin of stability (0.20 m). Consequently, the shoulder extends in order to pull the box toward body and increase the stability of the system. The shoulder extension starts from 5th time sequence and finishes to sequence 8. Likewise, in Fig. 9, we see that during the same time interval the stability increases (by decreasing TMA value from 0.15 to 0.08 m). In the next step (sequence 8–10) the shoulder flexion lifts the box up to the final position. However, the final positioning of box is also assisted by continues flexion of the elbow from sequence 5 to 10 (Fig. 7).

## Conclusion

Using different objective functions has enabled algorithm to simulate lifting motion with different dynamics. The presented results in Fig. 6 prove that there’s no collision for all predicted motions. So, the algorithm is robust to changes in dynamics of motion. The outcomes can be categorized as two distinguished lifting techniques i.e. squat lift and leg lift. It means algorithm is capable to generate two different motions which verify the generalization capability. The analysis carried out on the predicted angles and torques profiles of the joints shows that the kinematical results are consistent with the dynamical results. The outcome motions of the algorithm also were kinematically validated with the experimental results which demonstrate the result’s feasibility. What the collision avoidance algorithm does practically is to move the wrist away from the body in order to avoid the collision. While the wrist displacement could endangered the stability of the system, but all of the four predicted motions are stable (Fig. 9). In other words, the self-modeling approach successfully prevents the collision of the box with the body disregarding the dynamics of the movement while also guarantees its stability and reality. It also shows its kinematic and dynamic consistency with the optimal motion planning framework. Briefly, the design of the self-modeling algorithm and its integration in optimal motion planning framework has successfully represented through proving the robustness and generalization capability of the algorithm together with the stability and feasibility of the outcomes.
